# Association of Peripapillary Retinal Nerve Fibre Layer Thickness with Disability and MRI Findings in Multiple Sclerosis: A Retrospective Single-Centre Cohort Study

**DOI:** 10.3390/medicina62050904

**Published:** 2026-05-07

**Authors:** Ieva Vienažindytė, Kristė Kaikarytė, Vytautas Danielius, Ainė Žygaitė, Rasa Liutkevičienė, Renata Balnytė

**Affiliations:** 1 Department of Neurology, Medical Academy, Lithuanian University of Health Sciences, Eiveniu 2, LT-50161 Kaunas, Lithuania; ieva.vienazindyte@lsmu.lt (I.V.);; 2 Laboratory of Ophthalmology, Neuroscience Institute, Medical Academy, Lithuanian University of Health Sciences, Eiveniu 2, LT-50161 Kaunas, Lithuania; kriste.kaikaryte@lsmu.lt (K.K.); rasa.liutkeviciene@lsmu.lt (R.L.); 3 Medical Faculty, Medical Academy, Lithuanian University of Health Sciences, Eiveniu 2, LT-50161 Kaunas, Lithuania; aine.zygaite@stud.lsmu.lt; 4 Department of Ophthalmology, Medical Academy, Lithuanian University of Health Sciences, Eiveniu 2, LT-50161 Kaunas, Lithuania

**Keywords:** multiple sclerosis, optical coherence tomography, peripapillary retinal nerve fibre layer thickness, EDSS, Expanded Disability Status Scale, disability

## Abstract

*Background and Objectives:* The goal of this study was to evaluate the associations between peripapillary retinal nerve fibre layer (pRNFL) thickness, disability-related clinical measures, including the Expanded Disability Status Scale (EDSS), and report-based magnetic resonance imaging (MRI) findings in patients with multiple sclerosis (MS), and to explore potential longitudinal relationships between pRNFL changes and disability progression. *Materials and Methods:* A retrospective single-centre study was conducted in patients with MS diagnosed according to the 2010/2017 McDonald criteria at the Neurology Clinic of the Hospital of Lithuanian University of Health Sciences Kauno Klinikos. The study included 84 patients. pRNFL thickness was measured using optical coherence tomography (OCT) at baseline (defined as the time of diagnosis) and, for some patients, follow-up. Associations between pRNFL measures and clinical as well as MRI-derived variables were assessed using Spearman correlation and multivariable linear and ordinal regression analyses. *Results:* In cross-sectional analyses, lower baseline pRNFL thickness was associated with higher baseline disability (ρ = −0.257, *p* = 0.019) and greater worsening of EDSS over time (ρ = −0.268, *p* = 0.013). Significant associations were also observed between thinner pRNFL and pyramidal system impairment and bowel/bladder dysfunction. In adjusted linear regression models, each 20 µm reduction in pRNFL was associated with a 0.46-point increase in EDSS (B = −0.023, SE = 0.009) and a 0.32-point rise in cerebellar functional system score (B = −0.016, SE = 0.006). Among the 45 patients with repeat OCT, exploratory longitudinal pRNFL thinning showed directional trends toward increasing disability; however, these associations were not consistently significant after adjustment. Baseline pRNFL thickness showed a modest association with a composite MRI regional lesion score derived from report-based binary variables. *Conclusions:* In this real-world retrospective cohort, thinner pRNFL was associated with greater disability in cross-sectional analyses. Associations with individual MRI regions and radiological activity were limited and inconsistent. These findings should be interpreted as preliminary and hypothesis-generating. Further prospective studies are needed to clarify the potential role of OCT-derived pRNFL measurements.

## 1. Introduction

The course of multiple sclerosis is difficult to predict, making accurate prognosis and individualized treatment challenging [1]. Disease progression in multiple sclerosis (MS) is monitored by neurologists through assessment of patient-reported symptoms, neurological examination, disability scoring using the Expanded Disability Status Scale (EDSS), and longitudinal neuroradiological evaluation. Although patient-reported symptoms and objective examination play a crucial role in assessing the patient’s condition, they are relatively subjective and time-consuming; meanwhile, frequent repetition of imaging is expensive and, in some cases, contraindicated (e.g., claustrophobia, or adverse reaction to intravenous contrast agent, or certain implants). The discovery of additional biomarkers to predict the clinical course and future disability of multiple sclerosis would improve disease control and potentially provide the opportunity to monitor disease progression in a noninvasive, relatively inexpensive, and easily reproducible manner [2].

The retina of most patients with multiple sclerosis may show signs of both inflammatory and neurodegenerative processes [3], therefore optical coherence tomography (OCT) is used to assess these processes. OCT is a noninvasive, non-contact imaging method that uses near-infrared light to evaluate various structures of the eye. The thickness of the peripapillary retinal nerve fibre layer (pRNFL) and the ganglion cell layer (GCL) are among the most commonly measured OCT parameters for assessing optic nerve involvement. One of the main mechanisms underlying neurodegeneration in the visual system, including the optic nerve and visual pathways, is anterograde and retrograde axonal degeneration. In the context of retrograde optic nerve degeneration, demyelination of optic nerve axons leads to retrograde degeneration of the pRNFL and subsequently to degeneration of the GCL of the macula. Although retinal atrophy was initially thought to be caused solely by degeneration (mainly retrograde) [4,5], subsequent studies of retinal tissue from MS patients revealed several other pathological changes, including axonal loss, shrinkage of neuronal cell bodies, loss of synapses, microglial activation, and inflammation [3]. Macular GCL thickness measurements also help assess neurodegeneration in MS and may be more specific than pRNFL measurements [6].

Optic neuritis (ON), a common clinical feature of MS, is characterized by acute loss of visual acuity, pain on eye movement, and colour discrimination, and some patients are left with irreversible visual impairment due to permanent axonal damage [7]. OCT studies reveal that the pRNFL layer of the eyes of MS patients is significantly thinner than in healthy controls, and the degree of thinning correlates with disease progression and neurological disability as measured by the EDSS, even in the absence of recent ON episodes [8]. Studies have shown that pRNFL atrophy is most pronounced in the superior and nasal segments, associated with regional dynamics of myelin loss and axonal degeneration [9]. Studies of OCT dynamics have estimated that the thickness of pRNFL can undergo thinning by an average of 2–5 µm over several years, and more detailed analysis has shown that the rate of pRNFL thinning correlates with CNS atrophy in MRI data [10]. Recent molecular pathology studies reveal microglial activation, complement system-mediated axonal injury, and oligodendrocyte apoptosis as the main mechanisms leading to long-term degradation of the pRNFL [11]. For these reasons, the pRNFL assessment by OCT has the potential not only to be a diagnostic tool in ON, but also a noninvasive biomarker useful for the comprehensive assessment of neurodegeneration and disease progression in MS patients [12].

Multiple sclerosis can be associated with thinning of the optic nerves, even in people without optic neuritis, including those with benign MS. Optic nerve thinning can be objectively assessed by OCT [13]; thinning of the pRNFL has been described in the scientific literature even in patients with benign multiple sclerosis [14]. As previous studies have shown that these OCT findings are associated with multiple sclerosis-induced disability [15,16] and brain atrophy [17], they may serve as complementary biomarkers to brain imaging in predicting the disease course of multiple sclerosis.

Therefore, the aim of this study was to evaluate the associations between pRNFL thickness and clinical as well as radiological findings in patients with multiple sclerosis, as such analyses have not yet been performed in Lithuania.

## 2. Materials and Methods

### 2.1. Patient Data Collection

This retrospective single-centre observational study was conducted at the Neurology Department and Outpatient Clinic of the Hospital of Lithuanian University of Health Sciences Kauno Klinikos (LUHS Hospital KK). Patients included in the study had been diagnosed with multiple sclerosis according to the McDonald diagnostic criteria (2010 and/or 2017 revisions, as applicable at the time of diagnosis).

Medical data were retrospectively collected from the records of the Multiple Sclerosis Centre of the Neurology Clinic, the electronic hospital information system, and the hospital imaging archive (MedDream (Softneta, Vilnius, Lithuania)). The following variables were collected: demographic characteristics (age and sex), disease-related characteristics (ICD-10-AM diagnosis and disease course), disability measures based on the Expanded Disability Status Scale (EDSS), and neurological functional system scores based on the EDSS functional systems (visual, pyramidal, sensory, brainstem, cerebellar, cerebral/cognitive, and bowel/bladder function). Walking impairment was additionally categorized using EDSS-derived ambulation thresholds (e.g., EDSS > 4.5 indicating limitation to approximately 300 m walking distance and EDSS 5.0 indicating limitation to approximately 200 m). Paraclinical data included brain and/or spinal cord magnetic resonance imaging (MRI) reports and optical coherence tomography (OCT) examination reports. Whenever available, data from two time points were collected for comparison: baseline, defined as the time of diagnosis, and the last available follow-up assessment.

Medical records of 125 patients treated between 2020 and 2025 were screened for eligibility. Patients were included if clinical status was documented, EDSS had been assessed at least twice, and both brain MRI and OCT data were available. Thirty patients were excluded because of insufficient documentation, including missing relevant clinical data, unavailable EDSS assessment, missing brain MRI description, or unavailable/insufficient pRNFL measurements. In addition, OCT data from 11 patients were excluded because of ocular comorbidities that could substantially affect OCT measurements, such as ischemic optic neuropathy, diabetic retinopathy, retinal detachment, advanced cataract, or vitreous haemorrhage. The final analytical cohort consisted of 84 patients.

OCT imaging was performed using the Triton DRI OCT (Topcon Corporation, Tokyo, Japan), a swept-source OCT device operating at a wavelength of 1050 nm. pRNFL thickness measurements were obtained using the device’s standard optic disc scanning protocol centred on the optic nerve head, with automated segmentation provided by the manufacturer’s software. All scans were reviewed prior to inclusion in the analysis. Images with insufficient signal strength, motion artifacts, decentration, or segmentation errors were excluded. Although formal OSCAR-IB scoring was not prospectively recorded, all scans were reviewed using the same core quality principles commonly applied in OCT studies in neurological disorders, including assessment of signal strength, correct centration, segmentation accuracy, and absence of motion artifacts or other imaging artifacts. Only scans fulfilling the manufacturer’s recommended quality standards and judged to be of sufficient quality for analysis were included.

Optic neuritis (ON) was defined exclusively on the basis of clinically documented ON in the medical records. OCT-derived pRNFL values were not used to define, reclassify, or infer ON status. To reduce confounding by prior unilateral ON, a subject-level pRNFL variable was derived as the arithmetic mean of both eyes when neither eye had documented ON, or as the pRNFL value of the fellow eye without documented ON when unilateral ON had been documented. This approach is in line with prior OCT studies in multiple sclerosis [18,19,20]. In patients with bilateral ON, pRNFL data were retained for descriptive purposes, but such cases were interpreted with particular caution in analyses using subject-level pRNFL summary measures.

Baseline clinical assessment referred to the neurological evaluation performed at the time of diagnosis. OCT and MRI examinations were considered baseline examinations when they had been performed as part of the diagnostic work-up or within the corresponding diagnostic assessment period.

A subgroup of patients (*n* = 45, 54.6%) underwent at least two OCT examinations which allowed exploratory longitudinal analysis of pRNFL change over time in relation to changes in clinical disability measures.

In patients with a single OCT examination, the most recent neurological assessment and MRI report were used, and follow-up duration was calculated as the time from diagnosis to the latest available clinical evaluation. In patients with repeated OCT examinations, baseline and follow-up clinical assessments and MRI data were aligned as closely as possible to the timing of OCT examinations based on retrospective record availability. To ensure a uniform assessment for all patients, MRI findings were categorized according to lesion presence across four predefined anatomical regions consistent with routine multiple sclerosis reporting practice: periventricular, juxtacortical/subcortical, infratentorial, and spinal cord. Signs of radiological activity were recorded when contrast enhancement was described in the MRI report. MRI assessment was therefore based on report-based regional lesion presence rather than lesion counts or volumetric measurements. In addition, a composite MRI lesion score was derived by summing the number of involved regions (range 0–4).

Data on disease duration from symptom onset, relapse status at the time of OCT/MRI, corticosteroid use, and prior treatment of optic neuritis were not consistently available in the retrospective records and were therefore not included in the main multivariable analyses. Oligoclonal band status and disease-modifying treatment data were available but were not included in the main regression models because the present analyses were predefined as exploratory, and the sample size was limited for adding further covariates.

### 2.2. Statistical Analysis

Statistical analyses were performed using Microsoft Excel and IBM SPSS Statistics (version 30.0.0; IBM Corp., Armonk, NY, USA). Normality was assessed using the Shapiro–Wilk test. Because EDSS, EDSS change, and EDSS functional system scores are ordinal or semi-quantitative measures, correlations involving these outcomes were assessed using Spearman’s rank correlation coefficient (ρ).

Given the number of analyses performed, the study was considered exploratory and hypothesis-generating, and results were interpreted cautiously.

Multiple linear regression models were used to evaluate associations between OCT-derived pRNFL measures and disability-related outcomes. Separate cross-sectional models were constructed using baseline pRNFL thickness, whereas longitudinal models used change in pRNFL thickness. Depending on the outcome analysed, covariates included age, sex, disease course, and follow-up duration. Model performance was summarized using R^2^ or adjusted R^2^, as appropriate.

To evaluate multicollinearity, the variance inflation factor (VIF) was calculated for predictors included in the regression models. A VIF value < 5 was considered acceptable. All included predictors showed VIF values below this threshold, indicating no relevant multicollinearity.

An a priori power analysis was performed using G*Power (version 3.1.9.7). For a linear regression model with five predictors, assuming a medium effect size (f^2^ = 0.15), alpha = 0.05, and power = 0.80, the estimated required sample size was 92 participants. The available baseline sample (*n* = 84) was therefore slightly below this target and may have been underpowered to detect smaller effects, particularly in longitudinal analyses. The subgroup with repeated OCT measurements (*n* = 45) was analysed as an exploratory longitudinal sample.

## 3. Results

### 3.1. Subject Demographic and Clinical Characteristics

This study included 27 (32.1%) men and 57 (67.9%) women, with a mean age of 34 ± 9 years.

The distribution of disease phenotypes was as follows: relapsing-remitting multiple sclerosis (RRMS), *n* = 77 (91.6%); primary progressive multiple sclerosis (PPMS), *n* = 5 (5.9%); clinically isolated syndrome (CIS), *n* = 1 (1.2%); and radiologically isolated syndrome (RIS), *n* = 1 (1.2%).

At baseline, pyramidal impairment was the most common functional system abnormality (76.2%), whereas visual impairment was present in 32.1% of patients. At the last follow-up, pyramidal impairment was more frequent (85.7%), visual impairment remained similar in frequency (30.1%), and cognitive impairment had increased to 15.5%. Additional data are presented in Table 1.

At diagnosis, the median EDSS score was 2 points (range 0–6 for men, 0–5.5 for women) (Table 1). At the last follow-up, the median score increased to 2.5 points (range: 1.5–6 in men, 0–6 in women). The mean interval between baseline and follow-up assessments was 1281 ± 1112 days.

Among the 45 patients with follow-up OCT data, 30 (66.7%) had no documented history of ON at baseline, and 32 (71.1%) remained free of documented ON at follow-up. Data are presented in Table 2.

### 3.2. Baseline and Longitudinal Associations of pRNFL Thickness with Disability Measures

In cross-sectional analysis, baseline pRNFL thickness was inversely correlated with baseline EDSS score (ρ = −0.257, *p* = 0.019), indicating that thinner pRNFL was associated with greater disability. Baseline pRNFL thickness was also inversely correlated with EDSS change over follow-up (ρ = −0.268, *p* = 0.013). Additional correlations were observed with specific functional systems: pyramidal system impairment (ρ = −0.352, *p* = 0.001) and bowel/bladder dysfunction (ρ = −0.238, *p* = 0.029).

Older age was positively correlated with baseline disability (ρ = 0.341, *p* = 0.001). Associations with visual and pyramidal functional system scores are presented in Table 3, with directions consistent with the reported correlation coefficients.

No statistically significant association was observed between baseline pRNFL thickness and disease course (data shown in the Supplementary Materials). In the age- and sex-adjusted cross-sectional regression model, lower baseline pRNFL thickness was significantly associated with greater EDSS change over follow-up (B = −0.023, *p* = 0.015), and the overall model was statistically significant (model *p* = 0.013). Based on this model, a 20 µm lower baseline pRNFL thickness corresponded to an estimated 0.46-point greater increase in EDSS.

In an age- and sex-adjusted regression model, lower baseline pRNFL thickness was significantly associated with higher cerebellar functional system scores (B = −0.016, *p* = 0.007; model *p* = 0.037). According to this model, a 20 µm lower baseline pRNFL thickness corresponded to an estimated 0.32-point higher cerebellar functional system score. In the corresponding model, change in bowel/bladder functional system score was significantly associated with sex (B = −0.257, *p* = 0.008), but not with baseline pRNFL thickness (*p* = 0.267) or age (*p* = 0.744). The overall model was statistically significant (R^2^ = 0.105, *p* = 0.031), indicating that sex explained a significant portion of the variation in changes in bowel/bladder function. Relevant regression results are presented in Table 4.

In the exploratory longitudinal model including pRNFL change over time, greater pRNFL thinning was not significantly associated with EDSS change after adjustment (B = −0.049, *p* = 0.067). Although the overall model reached statistical significance (model *p* = 0.030), the association between pRNFL change and EDSS change did not. In this longitudinal model, a 10 µm greater reduction in pRNFL thickness corresponded to an estimated 0.49-point increase in EDSS; however, this association did not reach conventional statistical significance.

In the longitudinal model for EDSS-derived walking impairment, change in pRNFL thickness was not significantly associated with the outcome (B = 0.007, *p* = 0.673), whereas disease course was significantly associated with worsening walking impairment (B = 3.664, *p* < 0.001).

Similarly, in the longitudinal model for sensory functional system change, pRNFL thinning was not significantly associated with the outcome (B = −0.013, *p* = 0.328). In contrast, disease course (B = −1.262, *p* = 0.024) and follow-up duration (B < 0.001, *p* = 0.034) were significantly associated with sensory score change, and the overall model was statistically significant (model *p* = 0.027). Statistically significant results are presented in Table 5.

Spearman’s bivariate correlation analyses and ordinal logistic regression were performed to evaluate the association between retinal structural changes and MRI activity. The results of the regression analyses, including odds ratios and confidence intervals are presented in Table 6 and Table 7. A weak correlation was observed between pRNFL change over time and the presence of new periventricular lesion involvement (ρ = 0.216, *p* = 0.049); given the exploratory nature of the analysis and multiple comparisons, this finding should be interpreted cautiously.

Because MRI findings were assessed as binary report-based regional variables, an additional composite MRI lesion score was created by summing the number of involved anatomical regions. Each region was scored as 0 or 1, yielding a total score ranging from 0 to 4.

Ordinal logistic regression was used to assess the association between baseline pRNFL thickness and the composite MRI regional lesion score, adjusted for age and sex. Lower baseline pRNFL thickness was significantly associated with a higher composite MRI regional lesion score (B = −0.052, SE = 0.023, *p* = 0.023). For each 1 µm lower pRNFL thickness, the odds of belonging to a higher MRI regional lesion category increased by approximately 5% (OR = 0.95; 95% CI, 0.91–0.99).

Female sex showed a borderline association with a higher composite MRI regional lesion score (B = 0.988, *p* = 0.053), whereas age was not significantly associated with this outcome (Table 8). These findings should be interpreted with caution given the coarse nature of MRI assessment and the retrospective design of the study.

## 4. Discussion

The aim of our study was to examine the associations between pRNFL thickness, disability-related measures, and report-based MRI findings across predefined anatomical regions while accounting for selected clinical covariates. In this retrospective single-centre cohort of 84 patients, cross-sectional associations between thinner pRNFL and greater disability were more consistent than longitudinal associations. The longitudinal findings derived from the repeated-OCT subgroup should be regarded as exploratory.

The retina is increasingly recognized as a potential “window into” central nervous system pathology in multiple sclerosis. Previous studies have shown that pRNFL thinning measured by OCT correlates with brain atrophy and disability as assessed by EDSS [21].

Baseline was defined at the time of diagnosis rather than symptom onset, and data on disease duration from symptom onset were not available. This represents a potential source of systematic bias, as both baseline pRNFL thickness and baseline EDSS may already reflect variable pre-diagnostic disease duration across patients.

The present study differs from much of the existing literature in that it reflects real-world data from a single tertiary centre in Lithuania, an underrepresented setting in the OCT-MS literature. In addition, the study examined both disability-related clinical measures and a simplified regional MRI assessment available from routine practice rather than advanced research imaging metrics. Accordingly, these findings are best interpreted as preliminary real-world evidence rather than definitive biomarker validation.

OCT-derived retinal parameters have been proposed as promising biomarkers for the early diagnosis of multiple sclerosis and for monitoring disease course. However, most published studies have relied on quantitative MRI metrics, including lesion volume or brain atrophy measures, and/or additional retinal parameters such as the ganglion cell-inner plexiform layer (GCIPL) [22]. MRI assessment in the present study was based on coarse, report-based binary regional lesion involvement and a derived composite regional score rather than quantitative measures, and should not be interpreted as equivalent to quantitative MRI metrics.

Prospective studies are considered the methodological gold standard; however, they often require considerable time and financial resources. In contrast, retrospective cohorts offer a practical approach for preliminary hypothesis testing, especially in settings where resources are limited. Although such designs are subject to certain drawbacks, such as selection bias and reduced generalizability, they remain a valuable tool for describing real-world clinical populations and providing region-specific insights in the absence of prospective data.

In this context, our study contributes to the limited data from Lithuania by showing that baseline pRNFL thickness was associated with MS-related disability in this cohort, in line with findings from international cohorts [21,22]. Longitudinal pRNFL thinning showed directional trends toward increasing disability, although these associations were not consistently retained in multivariable analyses. These associations may reflect cumulative neurodegenerative changes related to disease duration rather than an independent prognostic effect of pRNFL. Similarly, baseline pRNFL thickness showed a modest and potentially limited association with the composite MRI regional lesion score. Although other investigators have analysed pRNFL after a single OCT, only a few have examined long-term thinning in relation to simple indicators of MRI lesion presence, and not all studies have consistently accounted for potentially relevant covariates such as age, sex, oligoclonal band status, or MS subtype [20,21,23].

In the subgroup of 45 patients with repeated OCT assessments, progressive pRNFL thinning showed limited and inconsistent associations with disability change after covariate adjustment. Accordingly, these longitudinal findings should be interpreted as exploratory and insufficient to establish prognostic value.

The limitations of this study include the retrospective design, predominance of relapsing-remitting disease, the use of report-based and relatively coarse MRI measures (binary regional lesion presence and the derived composite MRI score), the relatively small number of patients with serial OCT measurements, and the exploratory nature of multiple statistical comparisons. Given the number of statistical tests performed, there is an increased risk of type I error, and findings—particularly those with *p*-values close to the significance threshold—should be interpreted with caution. In addition, several clinically relevant variables were not incorporated into the main multivariable models, including disease duration from symptom onset, relapse status, corticosteroid exposure, prior treatment of optic neuritis, as well as treatment-related and cerebrospinal fluid variables.

The proportion of patients with documented ON in our cohort appears plausible in the context of previous multiple sclerosis literature, although retrospective ascertainment from medical records may have led to underestimation of prior ON episodes. Published studies indicate that ON may represent the initial demyelinating manifestation in a substantial subset of patients with MS and may occur during the disease course in a large proportion of patients, depending on cohort definition and follow-up duration [24,25]. Despite these limitations, the present findings provide region-specific preliminary data and support further prospective investigation of OCT-derived pRNFL measurements using standardized MRI metrics, more comprehensive clinical covariates, and larger longitudinal cohorts.

## 5. Conclusions

In this retrospective, single-centre cohort, thinner pRNFL was associated with greater disability and selected functional system impairment in cross-sectional analyses. Longitudinal pRNFL thinning showed less consistent associations with disability change and should be interpreted as exploratory. A modest and potentially limited association was observed between baseline pRNFL thickness and a composite MRI regional lesion score based on binary report-based variables. These findings should be considered preliminary and exploratory. Further prospective studies are required to determine the potential role of OCT-derived pRNFL measurements.

## Figures and Tables

**Table 1:** Disability assessment (according to the Expanded Disability Status Scale) and frequency of functional system impairment in patients with multiple sclerosis.

	Time of Diagnosis	Last Visit
	Total	Total
EDSS median (range)	2 (0–6)	2.5 (0–6)
Functional system		
Visual, *n* (%)	27 (32.1%)	26 (30.1%)
Pyramidal, *n* (%)	64 (76.2%)	72 (85.7%)
Sensory, *n* (%)	32 (38.1%)	35 (41.6%)
Brainstem, *n* (%)	41 (48.8%)	52 (61.9%)
Cerebellar, *n* (%)	40 (47.6%)	51 (60.7%)
Cognitive, *n* (%)	6 (7.1%)	13 (15.5%)
Bowel/bladder, *n* (%)	6 (7.1%)	9 (10.7%)
Reduced walking distance, *n* (%)	4 (4.8%)	11 (13.1%)

EDSS—Expanded Disability Status Scale; *n*—number of patients. Number (*n*) and percentage (%) of patients with multiple sclerosis with impairment in the functional systems assessed using EDSS functional system scores.

**Table 2:** Frequency of optic neuritis and optical coherence tomography (OCT) findings in patients with multiple sclerosis.

	Total Patients	
OCT performed during follow-up, *n* (%)	45 (54.6%)	
Patients with history of optic neuritis at baseline, *n* (%)	Left	Right
	4 (4.8%)	11 (13.1%)
Patients with history of optic neuritis at last visit, *n* (%)	Left	Right
	10 (22.2%)	3 (6.7%)
Mean pRNFL thickness (µm) at baseline (±SD)	Left	Right
	92.77 ± 23.062	93.49 ± 13.28
Mean pRNFL thickness (µm) at last visit (±SD)	Left	Right
	86.76 ± 18.788	88.87 ± 15.078

ON history is presented by eye; percentages are calculated within the subgroup of patients with follow-up OCT data. OCT, optical coherence tomography; pRNFL, peripapillary retinal nerve fibre layer; *n*, number of patients.

**Table 3:** Associations of baseline peripapillary retinal nerve fibre layer thickness with disability and functional system impairment in MS patients (Spearman correlation).

	Baseline pRNFL	*p*-Value	Sex	*p*-Value	Age	*p*-Value
Sex	−0.009	0.933	-	-	0.057	0.592
Age	−0.186	0.089	0.057	0.592	-	-
EDSS at baseline	−0.257	0.019	−0.053	0.626	0.341	**0.001**
EDSS change	−0.268	0.013	−0.104	0.345	0.189	0.084
Reduced walking distance	0.011	0.923	0.129	0.234	0.198	0.068
Visual FS score	−0.056	0.613	0.199	0.066	−0.242	**0.025**
Pyramidal FS score	−0.352	0.001	0.004	0.969	0.235	**0.030**
Sensory FS score	−0.035	0.755	0.041	0.710	0.041	0.071
Brainstem FS score	0.011	0.918	−0.134	0.218	0.099	0.364
Cerebellar FS score	−0.185	0.092	−0.002	0.988	0.121	0.269
Cognitive FS score	−0.052	0.642	0.086	0.428	−0.019	0.858
Bowel/bladder FS score	−0.238	0.029	0.164	0.132	0.179	0.101

Spearman correlation coefficients (ρ) with *p*-values. Functional system (FS) impairments are presented using EDSS functional system scores, where higher values indicate greater impairment. Abbreviations: MS, multiple sclerosis; pRNFL, peripapillary retinal nerve fibre layer; EDSS, Expanded Disability Status Scale.

**Table 4:** Associations between baseline peripapillary retinal nerve fibre layer thickness and disability-related outcomes in patients with multiple sclerosis (linear regression analysis).

EDSS-FS Score	Variable	Coefficient (B)	Standard Error (SE)	*p*-Value	R^2^	Model *p*-Value
EDSS change	pRNFL thickness (µm)	−0.023	0.009	**0.015**	0.126	**0.013**
	Age	0.012	0.012	0.342		
	Sex	−0.338	0.232	0.148		
Walking distance (EDSS-derived measure)	pRNFL thickness (µm)	−0.023	0.013	0.079	0.125	**0.013**
	Age	0.033	0.017	0.058		
	Sex	−0.461	0.317	0.149		
Cerebellar FS score	pRNFL thickness (µm)	−0.016	0.006	**0.007**	0.100	**0.037**
	Age	−0.014	0.008	0.082		
	Sex	0.124	0.148	0.404		
Bowel/bladder FS score	pRNFL thickness (µm)	−0.004	0.004	0.267	0.105	**0.031**
	Age	0.002	0.005	0.744		
	Sex	−0.257	0.095	**0.008**		

Linear regression analysis was used to assess the association between pRNFL thickness and EDSS-FS scores. Coefficients (B), standard errors (SE), and *p*-values are presented. Higher EDSS-FS scores indicate greater disability. EDSS, Expanded Disability Status Scale. Full model outputs are provided in the Supplementary Materials.

**Table 5:** Associations between changes in peripapillary retinal nerve fibre layer thickness and disability-related outcomes in patients with multiple sclerosis (linear regression analysis).

EDSS-FS Score	Variable	Coefficient (B)	Standard Error (SE)	*p*-Value	R^2^	Model *p*-Value
EDSS change	Change in pRNFL thickness (µm)	−0.049	0.026	0.067	0.287	**0.030**
	Age	0.001	0.017	0.935		
	Sex	−0.467	0.359	0.201		
	Disease course	1.695	1.056	0.118		
	Follow-up duration	<0.001	0.000	**0.037**		
Walking distance (EDSS-derived measure)	Change in pRNFL thickness (µm)	0.007	0.016	0.673	0.595	**<0.001**
	Age	0.004	0.010	0.677		
	Sex	−0.415	0.225	0.074		
	Disease course	3.664	0.664	**<0.001**		
Sensory FS score	Change in pRNFL thickness (µm)	−0.013	0.013	0.328	0.301	**0.027**
	Age	0.004	0.008	0.672		
	Sex	−0.256	0.190	0.186		
	Disease course	−1.262	0.535	**0.024**		
	Follow-up duration	<0.001	0.000	**0.034**		

Linear regression analysis was used to assess the association between change in pRNFL thickness and EDSS-FS scores. Changes in functional system evaluations (e.g., reduced walking distance) are presented as differences between follow-up and baseline assessments (coded as −1, 0, or +1), where higher values indicate worsening of symptoms. The main models are summarized here, whereas the full set of analysed models is provided in the Supplementary Materials.

**Table 6:** Associations between MRI findings and baseline pRNFL thickness in a single OCT measurement.

Lesion Region	Spearman’s ρ Coefficient	Spearman Correlation *p*-Value	Odds Ratio	95% Confidence Interval	*p*-Value (OR)
Subcortical	−0.187	0.242	0.977	0.802–1.191	0.820
Periventricular	−0.254	0.109	0.930	0.747–1.158	0.517
Infratentorial	−0.281	0.075	0.880	0.741–1.046	0.147
Spinal cord	−0.140	0.383	1.017	0.857–1.208	0.842
Signs of activity	0.060	0.710	1.107	0.952–1.289	0.185

Spearman’s ρ coefficients and *p*-values were obtained from bivariate correlation analysis. Odds ratios (OR), 95% confidence intervals (CI), and corresponding *p*-values were derived from ordinal logistic regression. Baseline pRNFL thickness refers to the mean of both eyes if unaffected, or the fellow eye without documented ON in cases of unilateral ON.

**Table 7:** Associations between MRI findings and change in pRNFL thickness over time.

Lesion Region	Spearman’s ρ Coefficient	Spearman Correlation *p*-Value	Odds Ratio	95% Confidence Interval	*p*-Value (OR)
Subcortical	−0.075	0.499	0.830	0.633–1.090	0.180
Periventricular	0.216	0.049	1.381	0.837–2.277	0.206
Infratentorial	0.056	0.613	1.051	0.936–1.181	0.396
Spinal cord	−0.018	0.869	0.992	0.923–1.065	0.818
Signs of activity	0.078	0.481	1.029	0.971–1.092	0.818

Spearman’s ρ coefficients and *p*-values were obtained from bivariate correlation analysis. Odds ratios (OR), 95% confidence intervals (CI), and corresponding *p*-values were derived from ordinal logistic regression.

**Table 8:** Association between the composite MRI regional lesion score and baseline pRNFL thickness.

Variables	B	SE	*p*-Value	OR (Exp[B])	95% CI (OR)
Single OCT pRNFL	−0.052	0.023	0.023	0.95	[0.91, 0.99]
Sex	0.988	0.510	0.050	2.69	[1.00, 7.30]
Age	0.013	0.025	0.596	1.01	[0.96, 1.06]

Ordinal logistic regression predicting the composite MRI regional lesion score (dependent variable: MRI_total_1; higher values indicate involvement of a greater number of anatomical regions). SE, standard error; OR, odds ratio; CI, confidence interval.

## Data Availability

The data presented in this study are available on request from the corresponding author due to privacy and ethical reasons.

## Supplementary Materials

The following supporting information can be downloaded at: https://www.mdpi.com/article/10.3390/medicina62050904/s1, Figure S1. Scatter plot illustrating the association between baseline pRNFL thickness and baseline EDSS (Spearman correlation). The line represents a linear fit for visualization purposes. Figure S2. Scatter plot illustrating the association between baseline pRNFL thickness and EDSS change (Spearman correlation). The line represents a linear fit for visualization purposes. Table S1. Multivariable linear regression models assessing associations between baseline pRNFL thickness and disability-related outcomes. Table S2. Multivariable linear regression models assessing associations between change in pRNFL thickness and disability-related outcomes.

## Abbreviations

The following abbreviations are used in this manuscript:

EDSS	Expanded Disability Status Scale
GCL	Ganglion Cell Layer
MRI	Magnetic Resonance Imaging
MS	Multiple Sclerosis
OCT	Optical Coherence Tomography
ON	Optic Neuritis
pRNFL	Peripapillary Retinal Nerve Fiber Layer
