# Supplementary material for: Association of Peripapillary Retinal Nerve Fibre Layer Thickness with Disability and MRI Findings in Multiple Sclerosis: A Retrospective Single-Centre Cohort Study
Source: Medicina (Kaunas). 2026 May 7;62(5):904. doi: 10.3390/medicina62050904 (PMC13208747; doi:10.3390/medicina62050904)
Supplement: Supplementary file 1 [file medicina-62-00904-s001.zip › medicina-4224182-supplementary.pdf]

Supplementary Materials:

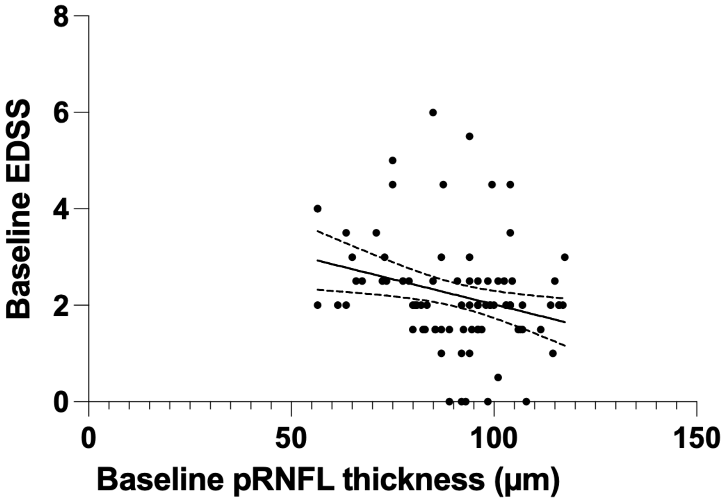

Figure S1. Scatter plot illustrating the association between baseline pRNFL thickness and baseline EDSS (Spearman correlation). The line represents a linear fit for visualization purposes.

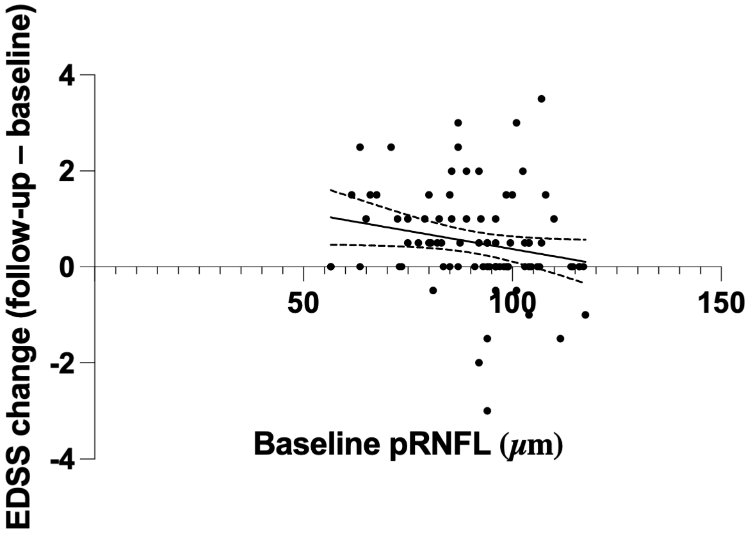

Figure S2. Scatter plot illustrating the association between baseline pRNFL thickness and EDSS change (Spearman correlation). The line represents a linear fit for visualization purposes.

Table S1. Multivariable linear regression models assessing associations between baseline pRNFL thickness and disability-related outcomes

EDSS-FS score	Variable	Coefficient (B)	Standard error (SE)	p-value	R^2	Model p-value
**EDSS change**	pRNFL thickness (µm)	-0.023	0.009	**0.015**	0.126	**0.013**
	Age	0.012	0.012	0.342		
	Sex	-0.338	0.232	0.148		
**Walking distance (EDSS-derived measure)**	pRNFL thickness (µm)	-0.023	0.013	0.079	0.125	**0.013**
	Age	0.033	0.017	0.058		
	Sex	-0.461	0.317	0.149		
**Visual FS score**	pRNFL thickness (µm)	-0.010	0.006	0.133	0.044	0.310
	Age	0.001	0.008	0.872		
	Sex	-0.140	0.155	0.370		
**Pyramidal FS score**	pRNFL thickness (µm)	0.001	0.007	0.842	0.001	0.995
	Age	0.000	0.009	0.969		
	Sex	0.025	0.166	0.881		
**Sensory FS score**	pRNFL thickness (µm)	-0.004	0.006	0.501	0.008	0.895
	Age	0.001	0.008	0.868		
	Sex	0.016	0.151	0.915		
**Brainstem FS score**	pRNFL thickness (µm)	0.000	0.007	0.956	0.026	0.544
	Age	0.009	0.010	0.328		
	Sex	0.172	0.179	0.340		
**Cerebellar FS score**	pRNFL thickness (µm)	-0.016	0.006	**0.007**	0.100	**0.037**
	Age	-0.014	0.008	0.082		
	Sex	0.124	0.148	0.404		
**Bowel/bladder FS score**	pRNFL thickness (µm)	-0.004	0.004	0.267	0.105	**0.031**
	Age	0.002	0.005	0.744		
	Sex	-0.257	0.095	**0.008**		

Linear regression models with baseline pRNFL thickness as the main predictor. Coefficients (B), standard errors (SE), p-values, R^2 values, and overall model p-values are presented. Models were adjusted for age and sex. Higher EDSS and EDSS functional system scores indicate greater disability. Abbreviations: pRNFL – peripapillary retinal nerve fibre layer; EDSS – Expanded Disability Status Scale.

Table S2. Multivariable linear regression models assessing associations between change in pRNFL thickness and disability-related outcomes

EDSS-FS score	Variable	Coefficient (B)	Standard error (SE)	p-value	R^2	Model p-value
**EDSS change**	Change in pRNFL thickness (μm)	-0.049	0.026	0.067	0.287	**0.030**
	Age	0.001	0.017	0.935		
	Sex	-0.467	0.359	0.201		
	Disease course	1.695	1.056	0.118		
	Follow-up duration	<0.001	0.000	**0.037**		
**Walking distance (EDSS-derived measure)**	Change in pRNFL thickness (μm)	0.007	0.016	0.673	0.595	**<0.001**
	Age	0.004	0.010	0.677		
	Sex	-0.415	0.225	0.074		
	Disease course	3.664	0.664	**<0.001**		
**Visual FS score**	Change in pRNFL thickness (μm)	-0.001	0.022	0.965	0.188	0.180
	Age	0.012	0.014	0.416		
	Sex	-0.697	0.308	**0.030**		
	Disease course	-0.712	0.907	0.438		
	Follow-up duration	<0.001	0.000	0.211		
**Pyramidal FS score**	Change in pRNFL thickness (μm)	-0.042	0.020	0.040	0.146	0.331
	Age	0.008	0.013	0.542		
	Sex	-0.139	0.272	0.611		
	Disease course	-0.286	0.800	0.723		
	Follow-up duration	<0.001	0.000	0.501		
**Sensory FS score**	Change in pRNFL thickness (μm)	-0.013	0.013	0.328	0.301	**0.027**
	Age	0.004	0.008	0.672		
	Sex	-0.256	0.190	0.186		
	Disease course	-1.262	0.535	**0.024**		
	Follow-up duration	<0.001	0.000	**0.034**		
**Brainstem FS score**	Change in pRNFL thickness (μm)	-0.046	0.019	**0.021**	0.167	0.248
	Age	0.001	0.012	0.937		
	Sex	0.108	0.265	0.685		
	Disease course	-0.043	0.780	0.957		
	Follow-up duration	<0.001	0.000	0.480		
**Cerebellar FS score**	Change in pRNFL thickness (μm)	0.007	0.017	0.687	0.037	0.926
	Age	-0.009	0.011	0.434		
	Sex	-0.162	0.239	0.502		
	Disease course	-0.188	0.704	0.791		
	Follow-up duration	<0.001	0.000	0.915		
**Cognitive FS score**	Change in pRNFL thickness (μm)	0.005	0.004	0.317	0.037	0.929

	Age	<0.001	0.003	0.872		
	Sex	0.033	0.062	0.603		
	Disease course	-0.003	0.183	0.989		
	Follow-up duration	<0.001	0.000	0.963		
**Bowel/bladder FS score**	Change in pRNFL thickness (μm)	0.004	0.009	0.636	0.168	0.242
	Age	0.006	0.006	0.350		
	Sex	-0.188	0.128	0.150		
	Disease course	-0.246	0.377	0.518		
	Follow-up duration	<0.001	0.000	0.104		

Linear regression models with change in pRNFL thickness as the main predictor. Coefficients (B), standard errors (SE), p-values, R^2 values, and overall model p-values are presented. Models were adjusted for age, sex, disease course, and follow-up duration. Changes in EDSS and functional system scores are expressed as differences between follow-up and baseline assessments. Higher values indicate worsening of symptoms.
